# Rest tremor revisited: Parkinson’s disease and other disorders

**DOI:** 10.1186/s40035-017-0086-4

**Published:** 2017-06-16

**Authors:** Wei Chen, Franziska Hopfner, Jos Steffen Becktepe, Günther Deuschl

**Affiliations:** 1 Department of Neurology, Shanghai Ninth People’s Hospital affiliated to Shanghai Jiao Tong University School of Medicine, 200011 Shanghai, China; 2 Department of Neurology, Universitätsklinikum Schleswig-Holstein, Kiel Campus, Christian-Albrechts-University, Rosalind Franklinstr.10, 24105 Kiel, Germany

**Keywords:** Tremor, Parkinson’s disease, Essential tremor, Dystonia, Pathophysiology

## Abstract

Tremor is the most common movement disorder characterized by a rhythmical, involuntary oscillatory movement of a body part. Since distinct diseases can cause similar tremor manifestations and vice-versa, it is challenging to make an accurate diagnosis. This applies particularly for tremor at rest. This entity was only rarely studied in the past, although a multitude of clinical studies on prevalence and clinical features of tremor in Parkinson’s disease (PD), essential tremor and dystonia, have been carried out. Monosymptomatic rest tremor has been further separated from tremor-dominated PD. Rest tremor is also found in dystonic tremor, essential tremor with a rest component, Holmes tremor and a few even rarer conditions. Dopamine transporter imaging and several electrophysiological methods provide additional clues for tremor differential diagnosis. New evidence from neuroimaging and electrophysiological studies has broadened our knowledge on the pathophysiology of Parkinsonian and non-Parkinsonian tremor. Large cohort studies are warranted in future to explore the nature course and biological basis of tremor in common tremor related disorders.

## Background

Tremor is defined as a rhythmical, involuntary oscillatory movement of a body part [1]. Making an accurate diagnosis of tremor disorders is challenging, since similar clinical entities may be caused by different diseases. Due to the lack of biomarkers, misdiagnoses among parkinsonian tremor, essential tremor (ET) and dystonic tremor are not uncommon [2]. Generally, tremor may be evaluated by features of medical history (family history, onset age, and temporal evolution), tremor characteristics (topography, activation condition, frequency and amplitude) and associated systemic or neurological signs. Besides, ancillary tests (dopamine transporter imaging, electrophysiological evaluation, response to levodopa etc.) are needed for patients with undetermined tremor entities (Table 1).

**Table 1:** Key clinical features and pathophysiological basis of tremor in Parkinson’s disease, essential tremor and dystonia

	Parkinsonian tremor	Essential tremor	Dystonic tremor
Key clinical features			
Topography	Hand > others	Hands > head > voice > others	head > Hands > others
Activation condition	Rest > postural/kinetic	Postural > kinetic > rest	Postural > kinetic > rest
Symmetry	Asymmetrical	Symmetrical	Asymmetrical
Suppression of tremor during movement onset	in most cases	not found	rare
Frequency	4-6Hz	4-8 Hz	7 Hz
Amplitude	Regular	Regular	Irregular
Potential accompanying signs	Bradykinesia, rigidity, etc	Impaired tandem gait	Dystonic posture
Possible pathophysiological basis			
Triggering factor	Dopaminergic dysfunction in nigrostriatal system	Reduced inhibition in cerebellum and brainstem	Reduced inhibitory reflex at multiple levels (spinal, brainstem, and cortical, etc.)
Activated circuit	Cerebello-thalamo-cortical circuit		

The consensus statement of Movement Disorder Society (MDS) on tremor in 1998 constitutes the main clinical classification system for tremor, is widely accepted and has been followed in the past two decades [1]. Many studies have been conducted to explore the prevalence and clinical correlates of tremor in common tremor related disorders. Some practical clinical cues and ancillary tests for clinical distinction are found [3]. Besides, accumulating structural and functional neuroimaging, as well as electrophysiological studies broaden our understanding on the pathophysiology of tremor in different kinds of movement disorders.

Therefore, the present review will mainly revisit the progress on prevalence and clinical features of rest tremor in Parkinson’s disease (PD), ET and dystonia. For patients with monosymptomatic tremor at rest, the possible clinical outcomes are discussed. Also, the potential ancillary tests for tremor differential diagnosis and underlying pathophysiological basis of tremor in common tremor related disorders are debated.

## Tremor in Parkinson’s disease

### Prevalence

The epidemiology of tremor in PD has not yet been deeply studied. A case series of 100 pathologically proven PD revealed tremor in 68% of cases at disease onset, in 75% during the course of the disease, and in 9%, tremor disappeared late in the course of the illness [4]. Rest tremor was noted at least during one evaluation among 47 pathologically verified Parkinsonian patients [5]. Action tremor was reported in 46–93% of PD patients depending on the selected populations [6, 7].

### Clinical features

All different forms of tremor i.e. rest, postural or kinetic tremor may occur in patients with PD. The most common form, classical Parkinsonian rest tremor, refers to a 4- to 6-Hz pill-rolling tremor in the fully resting limb, which is suppressed during movement initiation [8]. It can be provoked by stressful situations like backwards counting, tapping of the contralateral limb or by using the Stroop test. [9] The maximal amplitude is reached on average after 2–3 min [9]. As most motor symptoms of PD, rest tremor is often more pronounced unilaterally, and the upper limbs are usually more affected than the legs. Besides extremities, rest tremor also occurs in the tongue, lip or chin, but rarely involves the head. Other types like postural and kinetic tremor may also occur in PD. It is reported that action tremor is associated with rest tremor in PD, and appears more severely on the side of the body in which the rest tremor is predominant [6]. One of the most important diagnostic features is the suppression of rest tremor during movement onset [10]. Re-emergent tremor, defined as tremor slowly emerging when a new position of the extremity is acquired, is regarded as the same phenomenon when it is clinically more severely expressed [11].

Accumulating evidence indicates that tremor dominant PD is a distinct subtype from those with bradykinesia and rigidity. Clinically, tremor can even be worse on the opposite side of the more severe bradykinesia. Tremor dominant patients have higher dopamine transporter binding, compared to akinetic-rigid type [12], whereas tremor severity does not correlate with striatal dopamine deficits [13]. As demonstrated in DATATOP cohort, tremor dominant PD reported a less severe motor, cognition and functional disability than patients with postural instability and gait difficulty [14]. Prospective studies suggest that this subtype may predict a more favorable prognosis in terms of less mortality risk and lower probability of developing levodopa induced dyskinesia (LID) [15]. However, at later stages of the condition the course is similar for the akinetic-rigid variant and the tremor-dominant variant [16].

## Essential tremor

### Prevalence

The prevalence of ET ranges from 1% in the total population to 5% for those beyond the age of 60 years [17]. Regarding tremor localizations, arm tremor was reported in 90–100%, head tremor was reported in 21–44%; whereas voice tremor was found in 12–49% of ET. With respect to the conditions activating tremor, intention tremor (IT) occurs approximately in one third of ET cases [18]. Rest tremor was also noted in ET, its prevalence is highly dependent on the selected cohorts, ranging from as low as 2% in the population-based setting to 46% in the brain bank samples [19]. As a feature of ET, alcohol sensitivity was reported in at least 46% of the patients [20].

### Clinical features

ET is a common movement disorder defined by sparse clinical criteria. The clinical hallmark of ET is an involuntary postural or kinetic tremor affecting mainly the hands and forearms in the absence of other neurological signs, in particular dystonia and clear-cut ataxia or bradykinesia [1]. In addition to classic postural tremor in ET, patients may also have IT and rest tremor. Head and voice tremor are regarded as the second and third common tremor localizations [21].

Accumulating evidence indicates that ET is not a single entity but a group of diseases with diverse etiologies most of which are currently unknown [22, 23]. However, it is unknown if there are clinical criteria which separate different conditions. One promising stratifying marker is age at onset. Most epidemiological studies of ET show two distinct age at onset peaks: early and late disease onset. Early-onset patients more frequently reported a positive family history and alcohol sensitivity of tremor [24], whereas late-onset patients have faster tremor progression, more frequent dementia and earlier mortality [25]. Another subgrouping may be possible on the basis of the topography of ET. Phenotypic differences were found among patients stratified by head and voice tremor condition [21]. Female gender is associated with head tremor in ET as shown in our latest meta-analysis [21]. Both female and severe hand tremor may increase the risk of head and/or voice tremor in ET [21]. Activation conditions may also be phenotypical features separating different entities [26, 27]. It has been reported that ET patients with IT were older and had more frequent head and trunk involvement [26]; whereas patients with rest tremor were also older and have longer disease duration and greater tremor severity [19]. Neuroimaging studies reported that ET with head tremor had more cerebellar atrophy, especially in the vermis of the anterior lobe [28, 29]. Also, a recent pathological study found that patients with head and voice tremors had more Purkinje cell axonal swellings with torpedoes in the cerebellar vermis [30].

## Tremor in dystonia

Tremor has been recognized as an important clinical feature in dystonia. According to the consensus classification of tremor in 1998, tremor in dystonia may be classified into 1): dystonic tremor (DT), with tremor in the same body parts by dystonia, 2) tremor associated with dystonia (TAWD), with tremor in the unaffected regions of dystonia, and 3) dystonia gene-associated tremor [1].

### Prevalence

There is a wide variability of tremor prevalence in dystonia, ranging from 14 to 87%, due to selected cases and the sample size [31, 32]. So far, the largest case series found that 262 (55%) out of 473 adult-onset primary dystonia showed tremor as a main symptom, with head tremor being the most common affected location (*n* = 196, 41%), followed by hand tremor (*n* = 140, 30%) [33]. Among 140 patients with arm tremor, all presented postural tremor, 103 patients (73.6%) presented a kinetic component, whereas 57 patients (40.7%) also had rest tremor [33].

### Clinical features

Tremor occurs at, before or after the onset of dystonia. Tremor in dystonia is usually postural or kinetic but rest tremor can also be found [33]. Topographically, most studies reported a greater occurrence for head tremor than for upper limb tremor, whereas voice and leg tremor were considerably rare [31].

Accumulating observational studies indicated that some phenotypic parameters including gender, age at onset, localizations of dystonia, may influence the status of tremor in dystonia. Data on gender distribution in tremor of dystonia indicated a female to male predominance ranging from 1.2:1 to 3.7:1 [31]. Rest tremor was noted more frequent in patients with late-onset dystonia than in those with early-onset dystonia [34]. Based on two large cohorts with dystonia [33, 35], patients with segmental, multifocal and generalized dystonia had a higher proportion of tremor relative to focal dystonia. Among subgroups patients with focal dystonia, both studies showed a greater tremor occurrence in patients with cervical dystonia than in those with blepharospasm and task-specific upper limb dystonia.

Apart from the clinical characteristics, genetic factors may also contribute to the occurrence of tremor in dystonia. Pathogenic mutations in the anoctamin 3 gene (*ANO3*) were identified to cause autosomal dominant craniocervical dystonia and have been assigned to the dystonia locus dystonia-24 (DYT24) [36]. It was reported the presence of tremor was the characteristic feature in all affected individuals. In some individuals with *ANO3* mutations, tremor was the sole initial manifestation leading to the misdiagnosis of ET [37, 38]. Head tremor was also noted in DYT25 patients with guanine nucleotide-binding protein(*GNAL*) mutation [39].

## Monosymptomatic rest tremor (mRT)

A diagnostic challenge comes from patients presenting with predominant rest tremor without unequivocal bradykinesia or rigidity. According to MDS consensus statement on tremor in 1998, ‘monosymptomatic rest tremor’(mRT) was used if these patients had a tremor duration of at least 2 years [1]. Since rest tremor is a classic sign of PD, this entity of patients was also labelled as ‘benign tremulous Parkinsonism’(BTP) in some previous clinical studies [40, 41].

**Fig. 1:**
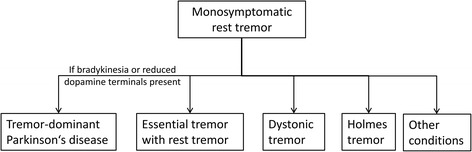
Diagnostic outcome of monosymptomatic rest tremor. (Deuschl G. Mov Disord.2013 [43])

There is still a debate on the etiology of mRT and its relation to PD. An early study indicated that patients with mRT had a nearly identical striatal dopaminergic deficit as in PD [42]. The majority of these patients with mRT will develop PD after a decade or more [43]. But they can also have ET with a rest component, dystonic tremor, Holmes tremor or a few even rarer conditions [43] (Fig. 1). If PD is the cause of mRT patients, this can be proven in-vivo with dopamine transporter (DAT) imaging. A recent prospective study found that 28 out of 33 patients with mRT developed PD verified by DAT positron emission tomography (DAT-PET), whereas 5 cases (15%) have scans without evidence of dopaminergic deficits (SWEDDs) [44]. An important pathological study came from 21 patients with the initial presentation of mRT (labeled by the authors as BTP). At postmortem, 16 of them fulfilled the neuropathological criteria of PD, 5 cases (24%) didn’t have nigral impairment and were diagnosed as ET with associated rest tremor or dystonic tremor [45]. These reports also provide a good argument for replacing the term ‘BTP’ by ‘mRT’ in the literature, as some of these cases never develop PD and if they do, the course is not benign [43].

## Ancillary tests for tremor differential diagnoses

The overlap among tremor disorders is wide and complex. ET patients may present postural tremor coexisting with resting tremor, while postural tremor may coexist with resting tremor in PD and tremor in dystonia is often mixed including a rest tremor component. For the most complicated tremor patients, Dopamine transporter imaging (DATScan) can provide objective evidence to demonstrate presynaptic nigrostriatal dopaminergic deficit in PD, whereas, it is normal in essential, dystonic and psychogenic tremor.

Several electrophysiological tests also showed the potential value for the clinical distinction [46] (Table 2). One method is based on quantified analysis of forearm electromyography (EMG) and accelerometry. Advanced mathematical techniques, in this case the ‘mean harmonic power’, using these data have shown to be a useful measure to separate clinically difficult cases of advanced ET from tremulous PD, and the accuracy is up to 94% [47]. As one of the most common functional movement disorders, psychogenic tremor (PT) can present with all kinds of tremor type with variable tremor frequency and severity. The key clinical features that help to differentiate psychogenic from organic tremor are a sudden tremor onset, unusual disease course, often with fluctuations or remissions, distractibility of the tremor if attention is removed from the affected body part, tremor entrainment, tremor variability, and a coactivation sign [48]. In cases where the clinical diagnosis remains challenging, providing a “laboratory-supported” level of certainty aids an early positive diagnosis [49]. Due to the inability of a patient with PT to generate voluntary tapping oscillations independent of their ongoing tremor oscillation, coherence entrainment test (CET) was used as a sensitive and specific means to distinguish PT from dystonic and other organic tremors, with nearly 100% concordance with clinical diagnosis [50]. Another method is blink reflex technique reflecting the brainstem excitability. Increased R2 recovery index was found in dystonic tremor, whereas it is within the normal range in ET, with 100% accuracy [51]. Regarding the interstimulus intervals (ISI) of 100 ms, this method also seems to be reliable (100% accuracy) for the distinction of Parkinsonian from essential tremor [52]. The third promising technique comes from sensory function tests. It is reported that temporal discrimination movement threshold (TDMT) is higher in ET, in contrast to dystonic tremor with increased somatosensory temporal discrimination threshold (TDT) [53]. Reduced reciprocal inhibition (RI) of arm muscles assessed with an H-reflex technique was found to be abnormal in patients with early onset of arm tremor and later development of torticollis, but ET had normal presynaptic inhibition [54]. Patients with a late and simultaneous onset of arm tremor and torticollis had normal RI [54]. This is put forward as an argument that dystonic tremor and tremor in dystonia do not necessarily share the same pathophysiological mechanism.

**Table 2:** Potential neurophysiological tests for tremor differential diagnosis

Techniques	Diagnostic reliability	Design	Parkinsonian tremor	Essential tremor	Dystonic tremor	Psychogenic tremor	References
Mean hormonic peak power	Accuracy 94%	Tremulous PD(*n* = 39) vs. ET(*n* = 41)	Tremulous PD > ET		-	-	Muthuraman M. Mov Disord. 2011 [47]
Coherence entrainment test (CET)	Accuracy 100%	Psychogenic trmeor (*n* = 10) vs. DT (*n* = 11) vs. HC(*n* = 10)	-	-	negative	positive	McAuley J. Mov Disord. 2004 [50]
Blink reflex recovery curve (BRrc)	Accuracy 100%	DT(*n* = 10) vs. ET(*n* = 10) vs. HC(*n* = 12)	-	Normal	Increased R2 recovery index	-	Nistico R. Neurology. 2012 [51]
	Accuracy 100%	tPD(*n* = 11) vs. ET with rest tremor(*n* = 10) vs. HC(*n* = 20)	Incresed R2 component	Normal at ISI 100	-	-	Nistico R. Parkinsonism Relat. Disord. 2014 [52]
Temporal discrimination movement threshold (TDMT)	PPV 86.7%, NPV 70.8%	ET(*n* = 19) vs. TAD(*n* = 20) VS. HC(*n* = 25)	-	Higher	Normal	-	Tinazzi M. Neurology. 2013 [53]
Somatosensory Temporal discrimination threshold (TDT)	PPV 100.0%, NPV 74.1%	TAD (*n* = 20) vs. ET(*n* = 19) VS. HC(*n* = 25)	-	Normal	Higher	-	Tinazzi M. Neurology. 2013 [53]
Reciprocal inhibition (RI)	Not given	CD with arm tremor(*n* = 13) vs. ET(*n* = 8)	-	Normal	Partially reduced	-	Munchau L. Brain. 2001 [54]

*TAD* Tremor associated with dystonia, *DT* dystonic tremor, *CD* cervical dystonia, *tPD* tremor dominant PD, *PPV* positive predictive value, *NPV* negative predictive value, *ISI* interstimulus intervals, -, not given

## Pathophysiology of Parkinsonian and non-Parkinsonian tremor

It is assumed that tremor networks within the brain are responsible for the different tremors. These circuits are not yet precisely known. Some nodes seem to play an important role. Ventral intermediate nucleus (Vim) of the thalamus is the relay site in cerebellar outflow pathway where deep brain stimulation (DBS) can improve almost all tremors (PD, ET and dystonia) indicating that cerebellum and its outflow pathway may involve in tremor genesis [55] (Table 1).

Emerging neuroimaging evidence showed that both the basal ganglia and the cerebellum are involved in Parkinsonian tremor [56]. It was reported that PD with rest tremor had more grey matter volume decrease in the right quadrangular lobe and declive of the cerebellum [57] and more iron accumulation in dentate nucleus, relative to those with akinetic-rigid type [58]. Data from functional neuroimaging indicates that dopaminergic dysfunction in pallidum triggers the onset of tremor, whereas, the tremor amplitude is regulated within the cerebello-thalamo-cortical circuit [59].

Theories on the pathophysiology of ET include the neurodegenerative, GABAergic and oscillatory network hypotheses [60]. For patients with ET, there is no postmortem gold standard for histopathological confirmation of the diagnosis, as it is in PD [61]. Limited pathological and neurochemical studies point to cerebellum and brainstem abnormalities in ET, including cerebellar Purkinje cell loss, axonal swellings (torpedoes) [62], as well as GABAergic dysfunction of the dentate nucleus in the cerebellum [63] and locus coeruleus in the brainstem [64]. The methodology beetwen different reseach groups investigating the pathology of ET diverge Torpedo cells, so-called Torpedoes were found only by one rearch group [62]. Other features like Purkinje cell loss in one study [65] cannot be confirmed by others [66]. Advanced neuroimaging provides an alternative way to understand the mechanism and networks involved in ET. A PET study indicated that ET is associated with reduced GABAergic function and increased binding of GABA receptor sites in brain regions implicated specifically in tremor genesis [67]. A recent study combining voxel-based morphometry, tractography and resting-state functional MRI suggests that a primary cerebellar defect leads to the emergence of a pathological oscillation which sets the tremor frequency, but the clinical manifestation of tremor is dependent on the cortical output [68].

Available electrophysiological studies demonstrate that patients with dystonia and tremor had reduced reciprocal inhibition between agonist and antagonist of upper limb muscles, a lack of brainstem interneuronal inhibition (BRrc), and abnormal sensory integration (TDT), indicating a lack of inhibitory mechanism at multiple levels (spinal, brainstem, and cortical) [31]. The neurophysiologic abnormalities in patients with dystonia and tremor resemble those in dystonia but differ from those described in ET, indicating tremor as phenotypic feature of dystonia. It has further been hypothesized that tremor in dystonia was caused by distorted cerebellar output due to abnormal burst firing pattern in Purkinje cells [31]. Structural MRI found that 27 (14%) of 188 dystonia cases had cerebellar atrophy or cerebellar lesions [69] whereas functional neuroimaging studies on tremor in dystonia are lacking.

## Conclusions

A series of epidemiological studies have yielded variable tremor prevalence among PD, ET and dystonia. These discrepancies may be partly due to sample selection as well as different definitions for rest tremor in parkinsonian tremor, ET and dystonic tremor and their subtypes. A diagnostic challenge comes from patients with mRT. Besides key clinical phenotypic differences and DAT scan, several transducer-based techniques like accelerometry, gyroscopy, EMG, and digitizing tablet-based meaures may provide extra clues for the distinction [70]. Compared to rating scales, these transducers are far more sensitive to changes in tremor amplitude and frequency. However, due to the natural variability of tremor, they are not more sensitive in defining the minimal detectable change than rating scales [70]. Also, their potential diagnostic values (sensitivity and specificity) still merit further validation in larger cohort studies. More studies on the pathophysiology of the different tremor entities are needed, which may help to develop new diagnostic markers and hence a more tailored therapeutic strategy. Studies on the natural course and biological basis of tremor are still warranted with standardized terminology, diagnostic criteria, validated evaluation tools and research protocols.

## Abbreviations

*ANO3*: Anoctamin 3 gene
BRrc: Brainstem interneuronal inhibition
BTP: Benign tremulous Parkinsonism
CET: Coherence entrainment test
DAT: Dopamine transporter
DBS: Deep brain stimulation
DT: Dystonic tremor
DYT24: Dystonia locus dystonia-24
EMG: Electromyography
ET: Essential tremor
*GNAL*: Guanine nucleotide-binding protein
IT: Intention tremor
LID: Levodopa induced dyskinesia
MDS: Movement disorder society
mRT: Monosymptomatic rest tremor
PD: Parkinson’s disease
PT: Psychogenic tremor
SWEDDs: Scans without evidence of dopaminergic deficits
TAWD: Tremor associated with dystonia
TDMT: Temporal discrimination movement threshold
TDT: Temporal discrimination threshold
Vim: Ventral intermediate nucleus
